# Tumeur neuroendocrine mammaire primitive: à propos d'un cas rare

**DOI:** 10.11604/pamj.2013.16.92.2531

**Published:** 2013-11-11

**Authors:** Kamilia Laabadi, Sofia Jayi, Aziza El Houari, Harmouch Tawfic, Hakima Bouguern, Hikmat Chaara, Abdilah Melhouf, Afaf Amarti

**Affiliations:** 1 Service de gynécologie obstétrique II, CHU Hassan II, Fès, Maroc; 2 Service d'anatomopathologie, CHU Hassan II, Fès, Maroc

**Keywords:** Carcinome neuroendocrine à grande cellule, cancer du sein, Large cell neuroendocrine carcinoma, breast cancer

## Abstract

Les carcinomes neuroendocrine primitifs du sein sont des tumeurs rares et représentent 2 à 5% des cancers mammaires. Nous rapportons le cas de localisation mammaire chez une patiente de 50 ans. Il s'agit d'une tumeur classée T4d N1 M0. La tumeur est suspecte radiologiquement. Une microbiopsie est réalisée. L’étude anatomopathologique et immunohistochimique est en faveur d'une tumeur neuroendocrine primitive du sein à grande cellules exprimant les récepteurs progestéroniques seulement. Vu le caractère inflammatoire de la tumeur une chimiothérapie est démarrée avec bonne évolution clinique. A la fin de la chimiothérapie on prévoit de réaliser une mastectomie avec curage axillaire et en fonction des résultats définitifs, une radiothérapie. Une hormonothérapie sera envisagée une 2ème étude immunohistochimique sur la pièce de mastectomie. Vu la rareté des carcinomes neuroendocrines mammaires primitifs, il n'existe pas de standard thérapeutique et le pronostic demeure difficile à déterminer.

## Introduction

Les carcinomes neuroendocrines sont Initialement décrits par Cubilla et al. En 1977, depuis d'autres cas ont été rapportés [1]. Les carcinomes neuroendocrines primitifs du sein sont des tumeurs rares [2]. Ils sont actuellement inclus dans la dernière classification de l'OMS des tumeurs du sein [3]. A partir d'un nouveau cas et une revue de la littérature nous discuterons les particularités anatomocliniques de cette entité rare et nous insisterons sur l'apport de l'immuno-histochimie dans le diagnostic.

### Patient et observation

**Figure 1:**
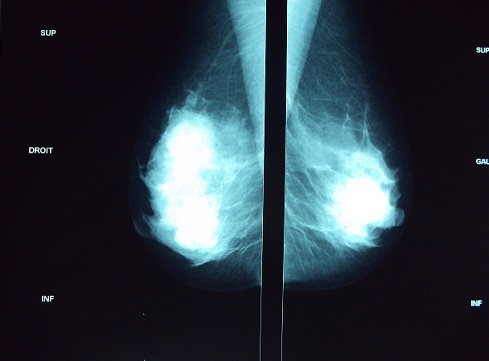
Cliché mammographique de profil montrant une opacité des quadrants supérieurs au niveau du sein droit, de contours polycyclique et partiellement bien limités avec des microcalcifications; lésion classée ACR4

**Figure 2:**
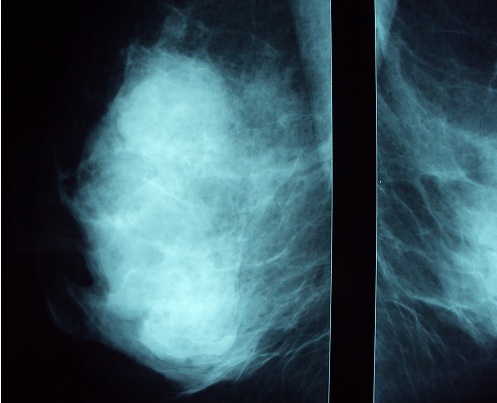
Agrandissement de l'opacité en cliché mammographique de profil du sein droit montrant mieux les caractéristiques sus décrites

**Figure 3:**
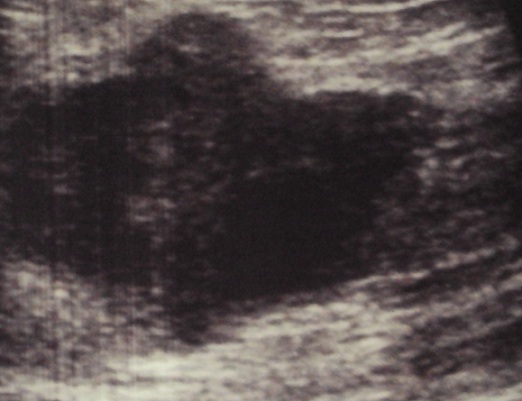
Aspect échographique montrant une masse tissulaire au niveau du quadrant supéro-externe du sein droit mesurant 42 mm, mal limitée; lésion classée ACR4

Il s'agit de Mme Z.C âgée de 50 ans, et qui présente depuis 2 ans un nodule du sein droit augmentant progressivement de volume. La patiente a bénéficié d'une mammographie objectivant une opacité au niveau du quadrant supéro-externe d'environ 4 cm de diamètre, de contours polycycliques et partiellement bien limités avec des microcalcifications classées ACR4 associés à un épaississement et rétraction cutanée en regard (Figure 1, Figure 2). L’échographie a objectivé la présence au niveau du quadrant supéro-externe d'une image tissulaire mal limité mesurant 42 mm classée ACR4 associé à deux adénopathies suspectes du creux axillaire droit mesurant 22 et 27 mm (Figure 3). Une indication d'une preuve histologique a été posée par son médecin traitant initialement mais la patiente s'est perdue de vue pour des raisons sociales et elle n'a consulté qu’à 2 ans après. Depuis un mois, l’évolution a été marquée par l'apparition de signes inflammatoires et une augmentation rapide de la taille tumorale d'où sa consultation dans notre formation pour prise en charge. Notre examen clinique a objectivé un gros sein droit avec une tumeur ulcéro-bourgeonnante prenant tout le sein (T4d N1 Mx).

**Figure 4:**
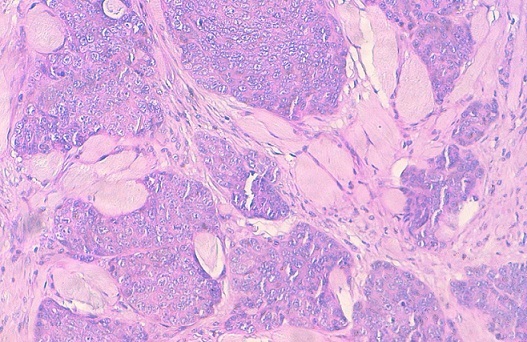
Aspect histologique montrant une prolifération carcinomateuse faite de massifs et de travées. Les cellules tumorales sont atypiques, munies d'un noyau irrégulier et hyperchromatique et d'un cytoplasme basophile. Les mitoses sont estimées à 30 mitoses/10CFG

La mammographie n'a pas pu être réalisé sur le sein droit car tout le sein droit est nécrosé et surinfecté. L’échographie mammaire a objectivé la présence d'une volumineuse masse tissulaire nécrosée occupant tout le sein droit accompagnée de poly adénopathies axillaires homolatérales mesurant de 30 à 40mm de diamètre. La microbiopsie au trucut a été réalisée et dont le résultat histologique est en faveur d'un carcinome neuroendocrine du sein droit sans composante in situ ni emboles vasculaires (Figure 4). Les récepteurs oestrogéniques sont négatifs, les récépteurs progestéroniques présente un marquage membranaire estimé à 2% alors que le KI67 est positif à 30%. L’étude immunohistochimique a été réalisée, les cellules tumorales expriment la chromagrannine, le CD56 et l'E-cadhérine et elles n'expriment pas la CK7 et la CK20. La TDM thoraco-abdominale ainsi que l’échographie cervicale étaient sans particularité et l'origine mammaire primitive a été retenue. La scintigraphie osseuse a mis en évidence une lésion osseuse suspecte de localisation secondaire du corps sternal.

La patiente a bénéficié d'une chimiothérapie. Elle est actuellement sous antracyclines et cyclophosphamides (3ème cure de chimiothérapie avec une bonne évolution clinique et une nette régression de la tumeur. On prévoit après la 6ème cure de chimiothérapie de réaliser une mastectomie droite et un curage axillaire homolatérale et en fonction des résultats histologique définitif compléter ou non par une radiothérapie. Une étude des récepteurs hormonaux sera refaite sur la pièce de mastectomie pour décider une hormonothérapie par la suite.

## Discussion

Les carcinomes neuroendocrines sont bien définis dans la classification de l'OMS 2003 par leur aspect morphologique similaire aux tumeurs neuroendocrines d'autres sites et par l'immuno-expression de marqueurs neuroendocrines dans plus de 50% du volume tumoral [1, 3]. Les carcinomes neuroendocrines primitifs du sein sont rares et représentent 2 à 5% des cancers mammaires [1–3]. Les formes primitives sont encore plus rares. La première description de tumeur neuroendocrine mammaire primitive est au profit de Wade et al. en 1983 [4, 5]. Depuis, une cinquantaine de cas a été rapportée et concernent uniquement la femme âgée. Le cas rapporté par Mecca et Busam est d'origine cutanée primitive et non mammaire primitive; c'est-à-dire que la tumeur est 100% neuroendocrine, développée à partir de la peau de la plaque aérolomamelonaire, avec envahissement de la glande mammaire [4]. Les tumeurs neuroendocrines s'observent essentiellement chez la femme âgée de 60-70 ans de la race blanche [1] mais des cas plus jeunes ont été rapportés dans la littérarture comme c'est le cas de notre patiente âgée de 50 ans de race blanche.

On décrit 4 groupes: les carcinomes neuroendocrines solides, les carcinoïdes atypiques, les carcinomes à petites cellules et les carcinomes à grandes cellules [1, 3]. Sapino et al. [6] ont proposé une classification en cinq types, rediscutant les classifications antérieurement proposées par Maluf et Koerner [7] et Papotti et al. [8], qui sont: la variante solide cohésive, la forme alvéolaire, la forme à petites cellules, la variante solide papillaire et le carcinome mucineux [1, 6]. Ces deux dernières formes se distinguent par la production du mucus et l'association fréquente d'un contingent in situ de type endocrine [6]. Dans notre observation, sur un petit échantillon (microbiopsie) on n'a pas objectivé de composante in situ. Mais il s'agit d'une tumeur neuroendocrine solide à grande cellules.

La tumeur neuroendocrine mammaire primitive est un diagnostic d'exclusion. L'octréoscan et le PET-scan éliminent les sites primitifs: poumons, sphère ORL, digestif, cutané [2, 9]. Aucun signe clinique n'est spécifique à ces tumeurs [10, 11]. Ces tumeurs se caractérisent par une évolution lente.et le motif de consultation le plus fréquent est un nodule du sein isolé ou associé à d'autres signes [10]. Notre cas vient appuyer les données de la littérature puisque notre patiente a présenté un nodule du sein ayant évolué sur 2 ans avant de présenter une poussée évolutive. Parfois ces tumeurs se manifestent par un placard érythémateux, violacé de la zone cutanée du sein bien limité [10]. Sur le plan radiologique, les opacités stellaires ou spéculées à la mammographie sont rares [12]. Ces tumeurs se présentent à la mammographie sous forme d'une masse dense aux contours irréguliers ou multilobulés, hypoéchogènes et homogènes à l’échographie [1], d'ailleurs, pour certains auteurs, cet aspect écho-mammographique est fortement évocateur d'une tumeur neuroendocrine [1]. La présence de microcalcifications est moins fréquente que dans les autres cancers mammaires. L'atteinte cutanée n'est observée que rarement, essentiellement dans les formes évoluées [10]. L'aspect écho- mammographique de notre patiente est celui décrit dans la littérature avec atteinte cutanée car la tumeur est à un stade évolué.

Le dosage des marqueurs biologiques (catécholamines urinaires, NSE, sérotonine) est peu contributif. La chromogranine A sérique a été, cependant, retrouvée à des concentrations plus élevées que celles de la normale dans deux cas de carcinomes neuroendocrines du sein rapportés par Sapino et al. [6]. En conséquence, plusieurs auteurs confirment que le dosage sérique de la chromogrannine A pourrait être un élément de surveillance des carcinomes neuroendocrines du sein [10, 13]. Il n'existe aucun examen qui permet d'orienter vers le carcinome neuroendocrine en dehors de l'examen histologique, qui seul permet de confirmer le diagnostic [10, 11]. Macroscopiquement, les carcinomes neuroendocrines primitifs du sein se présentent sous forme d'une tumeur ronde ou polylobée de couleur jaunâtre, de consistance ferme, ou rarement gélatineuse en cas de composante mucineuse associée [1, 3].

A l'histologie, le diagnostic de la nature neuroendocrine de ces tumeurs peut être suspectée à la morphologie et sera toujours confirmé après étude immuno-histochimique avec les marqueurs neuroendocrines, particulièrement la synaptophysine, le CD56 et la chromagranine A [1, 6]. L'immunohistochimie a remplacée les colorations à l'argent et la microscopie électronique. Dans la majorité des cas, la mise en évidence d'une sécrétion hormonale plus spécifique d'un siège tumoral n'est pas nécessaire au diagnostic [10]. Par ailleurs, la certitude de l'origine mammaire de ces tumeurs repose surtout sur la mise en évidence d'un contingent in situ; l'immuno-expression des récepteurs hormonaux par les cellules tumorales conforte également le caractère primitif ‘(mammaire de la tumeur [1, 14]. Par ailleurs, les récepteurs hormonaux sont rarement présents dans les carcinomes neuroendocrines du sein et rendre le pronostic plus favorable. Dans notre cas seuls Les récepteurs à la progestérone étaient présents comme dans le cas publié par Bourhaleb et al. [15]. Néanmoins, l'expression des récepteurs d'oestrogène et de progestérone dans les carcinomes neuroendocrines à grandes cellules du poumon et ceux d'autres sites a été rapportée. Leur expression dans le sein n'est pas donc la preuve bien déterminée de l'origine mammaire [15].

Dans cette observation, l'origine mammaire a été retenue après avoir éliminé une métastase tumorale d'autres sites par un bilan d'extension.

Le traitement des tumeurs neuroendocrines du sein est surtout chirurgical. Il combine: mastectomie, curage axillaire et métastasectomie. Les indications de la chimiothérapie et de la radiothérapie sont les mêmes que pour les autres cancers du sein. L'association d'une antiaromatase agit sur la composante mammaire. La composante neuroendocrine échappe généralement en quelques mois mais peut être contrôlée par une chimiothérapie à base d'anthracyclines [4]. L'hormonothérapie et l'immunothérapie ont un effet incertain. Les indications ne sont pas codifiées. Ces tumeurs répandent au traitement conventionnel des formes classiques de carcinomes mammaires et ne doivent actuellement pas faire l'objet d'une entité clinico-pathologique particulière [10].

L’évolution des tumeurs endocrines du sein est lente. Le pronostic des carcinomes neuroendocrines primitifs du sein dépend essentiellement du grade histologique et du stade anatomoclinique [1, 2, 14]. Ces tumeurs sont gradés histologiquement comme leurs homologues dans d'autres sites [1, 3]. Ainsi, les carcinomes neuroendocrines à variante solides et les carcinoïdes atypiques sont de meilleure pronostic que les carcinomes neuroendocrines à petites cellules et les carcinomes peu différencié à grandes cellules. La présence d'un contingent mucineux associé serait un facteur de bon pronostic [1, 6]. La survie à cinq ans dépasse 80%, toutes formes confondues. Cependant, les études récentes précisent la fréquence de récidives locorégionales et de métastases, ce qui rend le pronostic redoutable dans l'ensemble [6, 10, 16]. Les facteurs pronostiques admis sont représentés par l’âge, le terrain, le pouvoir de sécrétion de la tumeur, la taille tumorale et l'existence ou non de métastases [10, 16].

## Conclusion

Le carcinome neuroendocrine primitif du sein est une entité rare, d'individualisation récente et dont le pronostic est redoutable dans l'ensemble. Les études concernant cette entité sont rares et regroupent un effectif réduit de cas. L’étude de séries plus larges permettra de mieux connaitre leur histogenèse et leur profil évolutif et d'adapter le bilan d'extension et la stratégie thérapeutique.
